# The phenotype of vascular smooth muscle cells co-cultured with endothelial cells is modulated by PDGFR-β/IQGAP1 signaling in LPS-induced intravascular injury

**DOI:** 10.7150/ijms.34749

**Published:** 2019-08-06

**Authors:** Xia Zheng, Xiaotong Hu, Wang Zhang

**Affiliations:** 1 Department of Critical Care Medicine, The First Affiliated Hospital of Zhejiang University, Hangzhou, Zhejiang, 310003, P.R. China.; 2 Collaborative Innovation Center for Diagnosis and Treatment of Infectious Diseases; The First Affiliated Hospital, College of Medicine, Zhejiang University, Hangzhou, Zhejiang, 310003, P.R. China.

**Keywords:** sepsis, endothelial cells, smooth muscle cells, phenotype, Intravascular Injury.

## Abstract

**Background** Sepsis, a leading cause of death in intensive care units, is generally associated with vascular dysfunction. However, its pathophysiological process has not been fully clarified, lacking in-depth knowledge of its pathophysiological process may hinder the improvement of diagnosis and therapy for sepsis. Hence, as the key parts of the vascular wall, the interaction between endothelial cells (ECs) and smooth muscle cells (SMCs) under septic situation need to be further studied.

**Methods** ECs and SMCs were co-cultured using Transwell plates. Lipopolysaccharide (LPS) was used to induce sepsis. A scratch-wound assay was used to assess cell migration, and western blotting was used to assess the level of redifferentiation of SMCs as well as the expression of PDGFR-β and IQGAP1.

**Results** Co-culture with ECs reduced the redifferentiation of SMCs induced by LPS (10 μg/ml), which was characterized by increased migration ability and decreased expression of contractile proteins (e.g., SM22 and α-SMA). The production of TNF-α could decrease the level of PDGFR-β in SMCs. Treatment of SMCs with the PDGFR-β inhibitor imatinib (5 μM) was able to counteract LPS-induced SMC redifferentiation and reduce IQGAP1 protein expression, especially when SMCs were co-cultured with ECs.

**Conclusion** The phenotype of vascular SMCs co-cultured with ECs was modulated by IQGAP1 through the PDGFR-β pathway, which may lead to vascular remodeling and homeostasis in LPS-induced intravascular injury. This pathway could be a novel target for the treatment of vascular damage.

## Introduction

Sepsis is a major challenge in public health care. Considerable resources have been invested in developing potential therapies 1. The current treatment for sepsis is primarily symptomatic support 2. This is likely a result of the lack of in-depth knowledge of the underlying pathophysiological processes of sepsis. It is well known that vascular dysfunction is a decisive factor in the development of several inflammatory diseases. The mechanisms underlying septic induction of oxidative and nitrosative stresses, the functional consequences of these stresses, and potential adjunct therapies for microvascular dysfunction have been identified 3. As demonstrated in the literature, the interaction between endothelial cells (ECs) and smooth muscle cells (SMCs) is an essential maturation process in physiological conditions 4, 5. Generally, *in vitro* studies are based on single cell cultures, which exclude interactions between different cell types. Previous studies have suggested that ECs could regulate vascular tone through the release of vasoactive molecules such as platelet-derived growth factor (PDGF)-BB 6, 7 and tumor necrosis factor-α (TNF-α) 8. There are two types of SMCs: contractile and synthetic SMCs. Synthetic SMCs have stronger migratory ability compared with contractile SMCs 9. Contractile and synthetic SMCs can be distinguished by differences in the expression levels of marked proteins, such as smooth muscle 22 (SM22) and α-smooth muscle actin (α-SMA), which are known as contractile SMC proteins. Studies have shown that the PDGF receptor (PDGFR)-β pathway plays a key role in SMC phenotypic modulation by suppressing the expression of SM22 and α-SMA 10, 11, resulting in a synthetic phenotype that can facilitate the infiltration of inflammatory cells 12.

IQ-domain GTPase-activating protein 1 (IQGAP1) plays a key role in regulating cell migration 13-15. A previous study demonstrated that IQGAP1 expression was markedly increased in vascular diseases caused by complete removal of the endothelium 16, and that IQGAP1 played a critical role in SMC migration at least in part by increasing PDGFR in focal adhesions, as well as by increasing focal adhesion formation at the leading edge 16. However, the effects of IQGAP1 on SMC phenotypic transformation and migration following vascular damage caused by sepsis remain unknown. The present study investigated the role of the PDGFRβ/IQGAP1 pathway in EC-mediated SMC phenotypic transformation and migration during sepsis in a co-culture cell model.

## Methods

### Reagents

Lipopolysaccharide (LPS, Escherichia coli 055: B5, Cat. No. L2880; Sigma-Aldrich) was used to mimic a septic condition. LPS was diluted by phosphate-buffered saline (PBS); The chemical inhibitor imatinib mesylate (Cat. No. S1026; Selleck) was used to inhibit the PDGFR; The first antibodies included anti-IQGAP1 (1:1000; # ab86064, Abcam), anti-α-SMA (1:1000; #A5228; Sigma-Aldrich), anti-SM22 (1:1000; #ab137453; Abcam), anti-GAPDH (1:1000; #5174; Cell Signaling Technology); A horseradish peroxidase (HRP)-conjugated secondary antibody was purchased from Cell Signaling Technology (1:5000, #7074).

### Cell Culture and intervention

Human umbilical vein SMCs (Cat. No. 8020) and ECs (Cat. No. 8000) were purchased from ScienCell. SMCs were cultured in basal medium (SMCM, Cat. No.1101; ScienCell), supplemented with 2% fetal bovine serum (FBS, Cat. No. 0010; ScienCell), 1% smooth muscle cell growth supplement (SCGS, Cat.No.1152; ScienCell). ECs were cultured in basal medium (ECM, Cat. No.1001; ScienCell) containing 5% fetal bovine serum (FBS, Cat. No. 0025; ScienCell), and 1% endothelial cell growth supplement (ECGS, Cat. No. 1052; ScienCell). After 1% penicillin/streptomycin (P/S, Cat. No. 0503; ScienCell) was added, they were maintained at 37 °C in a humidified 5% CO_2_ incubator. Passages 3-8 were used for the experiments.

### Co-culture of ECs and SMCs

The co-culture system was established by using the Transwell plates (Cat. No. 3470; Corning)^17^, with SMCs were seeded in the lower wells and the ECs were planted in the transwell inserts. Before ECs were co-cultured with SMCs for 24 hours, they both were separately pretreated as follows: culturing with control vehicle (Control), culturing with LPS (LPS), culturing with LPS and imatinib (LPS + imatinib) according to different experiment design, imatinib was given before LPS for 90 min, then SMCs and ECs were extensively washed with PBS to remove excess LPS and/or imatinib which were not taken up. Serum-free mediums were added, in order to exclude any confounding factors contained in the serum.

### Enzyme-Linked immunosorbnent assay

Levels of PDGF-BB and TNF-α were determined in the supernatants of different groups using commercial high-sensitivity ELISAs, according to the manufacturer's instructions (Cat. No.EK91372, MultiSciences Biotech, Co., Ltd).

### Wound healing assay

SMCs were seeded in six-well plates according to different groups. The cell monolayer was scratched using a 200μl pipette tip before washing three times with PBS to clear cell debris and floating cells. One thousand microliters of serum-free SMCM was then added, and the cells were incubated for 24 h at 37 °C in 5% CO_2_. Images were captured under a microscope before and after the 24 h incubation at the same position. Migration ability was measured by calculating the rate of scratch wound confluence after 24 h using Adobe Photoshop 2016 software (Adobe Systems Inc.,).

### Western Blot Analysis

According to general procedure, western Blot Analysis was performed, briefly, Equal amounts of lysates of cells were applied to 4-12% SDS-PAGE precast gels (Cat. No. NP0335, Invitrogen; Thermo Fisher Scientific), Resolved proteins were transferred to polyvinylidene fluoride (PVDF) membranes (Cat. No. IPVH00010; Merck Millipore), blocked, and then incubated with the primary and second antibody, then the protein bands were visualized by enhanced chemiluminescence kit (Cat. No. 70-P1421; MultiSciences Biotech, Co., Ltd.,) and exposed to X-ray film. The expression of the protein was analyzed by Image J software.

### CCK-8 assay

Cells (1 × 10^5^ cells/ml) were grown in 96-well plates and then starved for 24 h before being subjected to treatment according to the experimental requirement, cells were then harvested and washed with PBS and cell counting kit-8 (CCK-8; Dojindo) mixed with FBS-free medium was used for cell viability assay.

## Statistical analysis

All results were shown as mean ± SD. Statistical significance was assessed by unpaired Student's t-test or ANOVA, P-values of 0.01 and 0.05 were considered significant. *p < 0.05, **p < 0.01.

## Results

### Co-culture of ECs and SMCs resulted in higher SMC TNF-α expression

**Figure 1:**
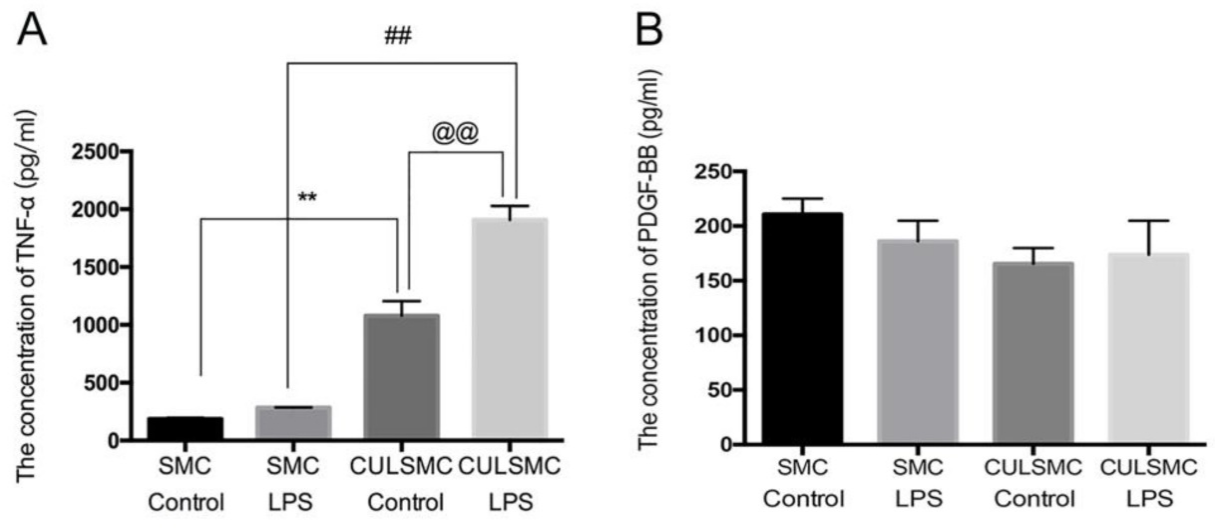
** ECs induced higher TNF-α expression in SMCs**. **(A)**, The quantification of TNF-α expression in SMCs under single-culture or co-culture system. **: P<0.01, SMCs were treated with control vehicle in single-culture system (SMC Control) vs SMCs were treated with control vehicle in co-culture system (CULSMC Control), ##: P<0.01, SMCs were treated with LPS in single-culture (SMC LPS) vs SMCs were treated with LPS in co-culture (CULSMC LPS), @@: P<0.01, CULSMC Control vs CULSMC LPS; **(B)**, No significant difference in the level of PDGF-BB was shown in the above-mentioned groups.

SMCs and ECs were treated according to the experimental design shown in Figure 1. The supernatants were collected from SMCs for an enzyme-linked immunosorbent assay (ELISA), which indicated a slight upward trend in the level of TNF-α in single-culture SMCs treated with lipopolysaccharide (LPS); there was no significant difference between the LPS-treated and control groups. However, in the co-culture system, ECs induced higher TNF-α expression in SMCs compared with single-culture SMCs with or without LPS treatment (mean ± standard deviation, 1077.37 ± 127.90 pg/ml vs. 187.47 ± 10.45 pg/ml; P < 0.01 without LPS treatment; 1907.69 ± 119.79 pg/ml vs. 284.17 ± 1.60 pg/ml; P < 0.01 with LPS treatment). When the co-culture system was stimulated with LPS, TNF-α expression reached the highest level of the four subgroups (Figure 1A). There were no statistical differences in the levels of PDGF-BB in the above-mentioned groups (Figure 1B).

### ECs affect the SMC phenotype in a paracrine manner

**Figure 2:**
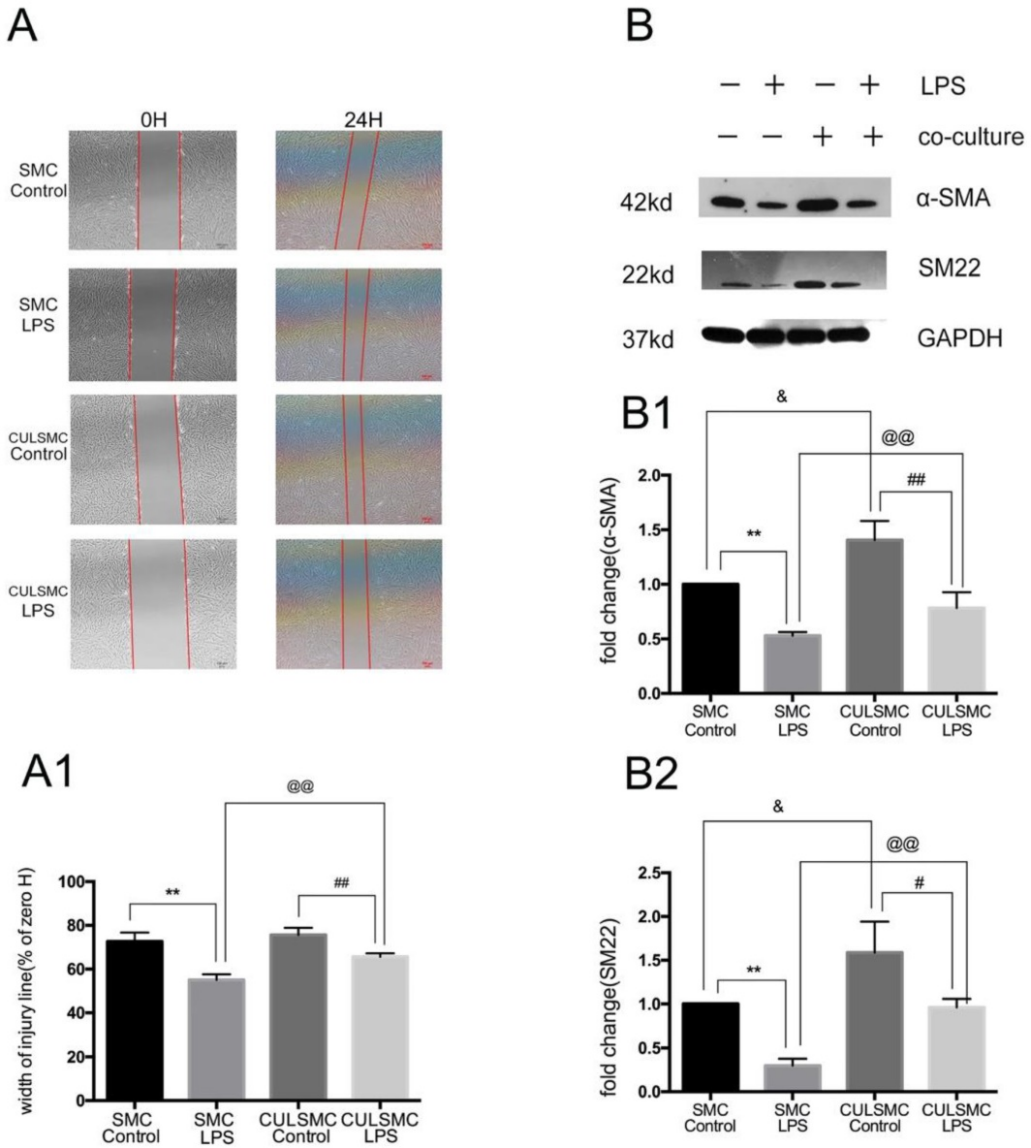
** SMCs Shift phenotypic transition to synthetic type from contractile type orchestrated by ECs. (A)**, LPS increased the migration ability of SMCs. When SMCs were co-cultured with ECs, the increased migration induced by LPS was alleviated. **(A1)**, Quantification of A is shown in A1, [% width of injury line = (a - b) × 100%/a; a = Initial scratch wound area at 0 h, b = Scratch wound area at 24 h] **: P<0.01, SMC Control *vs* SMC LPS, ##: P<0.01, CULSMC Control *vs* CULSMC LPS, @@: P<0.01, SMC LPS *vs* CULSMC LPS; **(B)**, LPS induced the reduction of SM22 and α-SMA in SMCs, however, when SMCs were co-cultured with ECs, the reduction of SM22 and α-SMA protein induced by LPS were partly alleviated. Meanwhile, ECs could enhance the expression of SM22 and α-SMA in SMCs in normal condition. **(B1 and B2)**. Quantification of B is shown in B1 and B2, **: P<0.01, SMC Control *vs* SMC LPS, ##: P<0.01, #: P<0.05, SMC LPS *vs* CULSMC LPS; CULSMC Control *vs* CULSMC LPS, @@: P<0.01, SMC LPS *vs* CULSMC LPS; &: P<0.05, SMC Control *vs* CULSMC Control.

To ascertain whether ECs can affect the SMC phenotype in a paracrine manner, single-culture SMCs were treated with control vehicle or LPS for 24 h. After the cell monolayer was scratched, serum-free medium was added to the culture and the cells were incubated for another 24 h. In the co-culture system, SMCs and ECs were treated with or without LPS separately for 24 h prior to co-culture, and the subsequent steps were performed as described above for the single-culture SMCs. The scratch-wound assay revealed that LPS increased the migration ability of SMCs compared with the control group in both the single- and co-culture systems. When SMCs were co-cultured with ECs, the increased migration induced by LPS was alleviated. ECs had no influence on the migration ability of SMCs in the absence of LPS (Figure 2A and 2A1).

Western blotting demonstrated that LPS treatment for 24 h eventually led to a phenotypic transition characterized by the reduction of SM22 and α-SMA in SMCs. When SMCs were co-cultured with ECs, the protein levels of SM22 and α-SMA were significantly increased; when treated with LPS, the levels of SM22 and α-SMA were somewhat decreased. Additionally, when exposed to LPS, SM22 and α-SMA protein expression in SMCs co-cultured with ECs was markedly higher compared with single-culture SMCs (Figure 2B).

**Figure 3:**
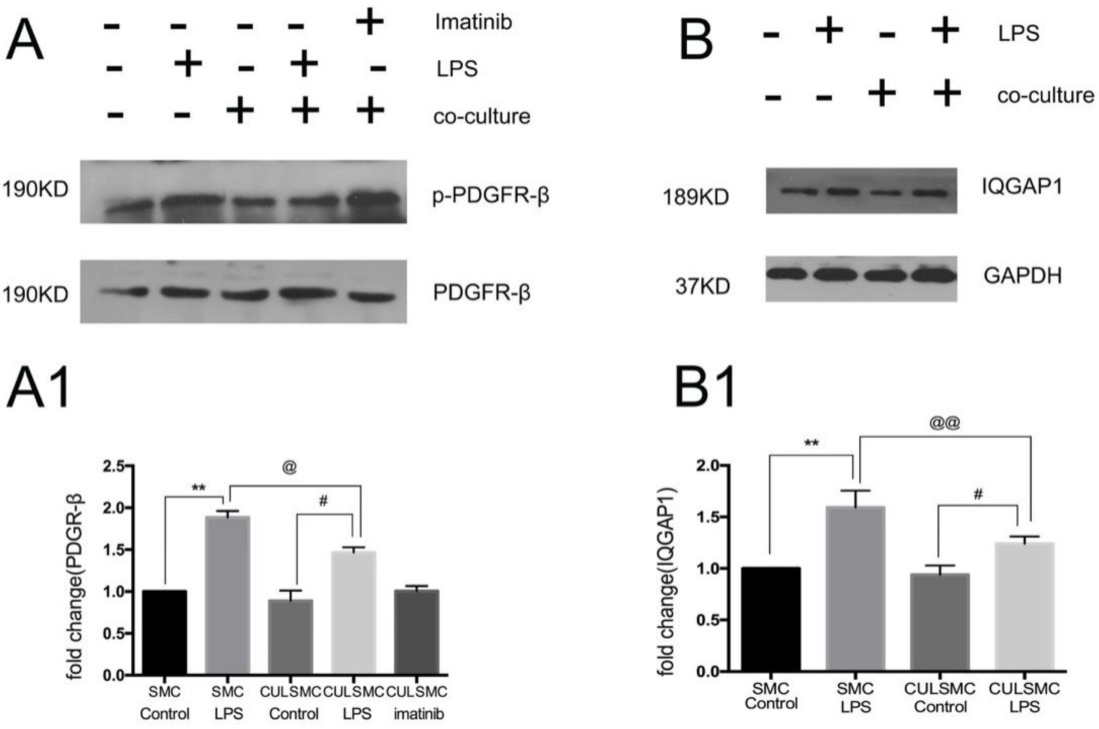
** LPS induced a higher higher level of PDGFR-β and IQGAP1 in SMCs in both the single and co-culture systems. (A)**, LPS induced the level of p-PDGFR-β expression in SMCs in both single and coculture system, but when SMCs were co-cultured with ECs, both control and LPS group experienced the relative lower p-PDGFR-β protein expression than that in single culture system. With LPS stimulation, p-PDGFR-β levels in co-culture SMCs were remarkably lower than those of single-culture SMCs. Quantification of A is shown in A1; **: P<0.01, SMC Control *vs* SMC LPS, #: P<0.05, CULSMC Control *vs* CULSMC LPS, @: P≤0.05, SMC LPS *vs* CULSMC LPS; **(B)**, the change of IQGAP1 expression was mediated by LPS, Quantification of B is shown in **(B1)**; **: P<0.01, SMC Control *vs* SMC LPS, #: P<0.05, CULSMC Control *vs* CULSMC LPS, @@: P<0.01, SMC LPS *vs* CULSMC LPS.

As shown in Figure 3A, when compared to control groups, LPS induced a higher level of phosphorylated PDGFR-β (p-PDGFR-β) in SMCs in both the single and co-culture systems. ECs induced a slight decrease in p-PDGFR-β levels of SMCs in the absence of LPS treatment, but this difference was not statistically significant. With LPS stimulation, p-PDGFR-β levels in co-culture SMCs were remarkably lower than those of single-culture SMCs.

### IQGAP1 is involved in SMC differentiation in the co-culture system

As illustrated by western blotting, increased expression of IQGAP1 was observed in LPS-treated SMCs in both the single and co-culture systems. In the co-culture system, the increased expression of IQGAP1 in LPS-treated SMCs was partly alleviated (Figure 3B).

**Figure 4:**
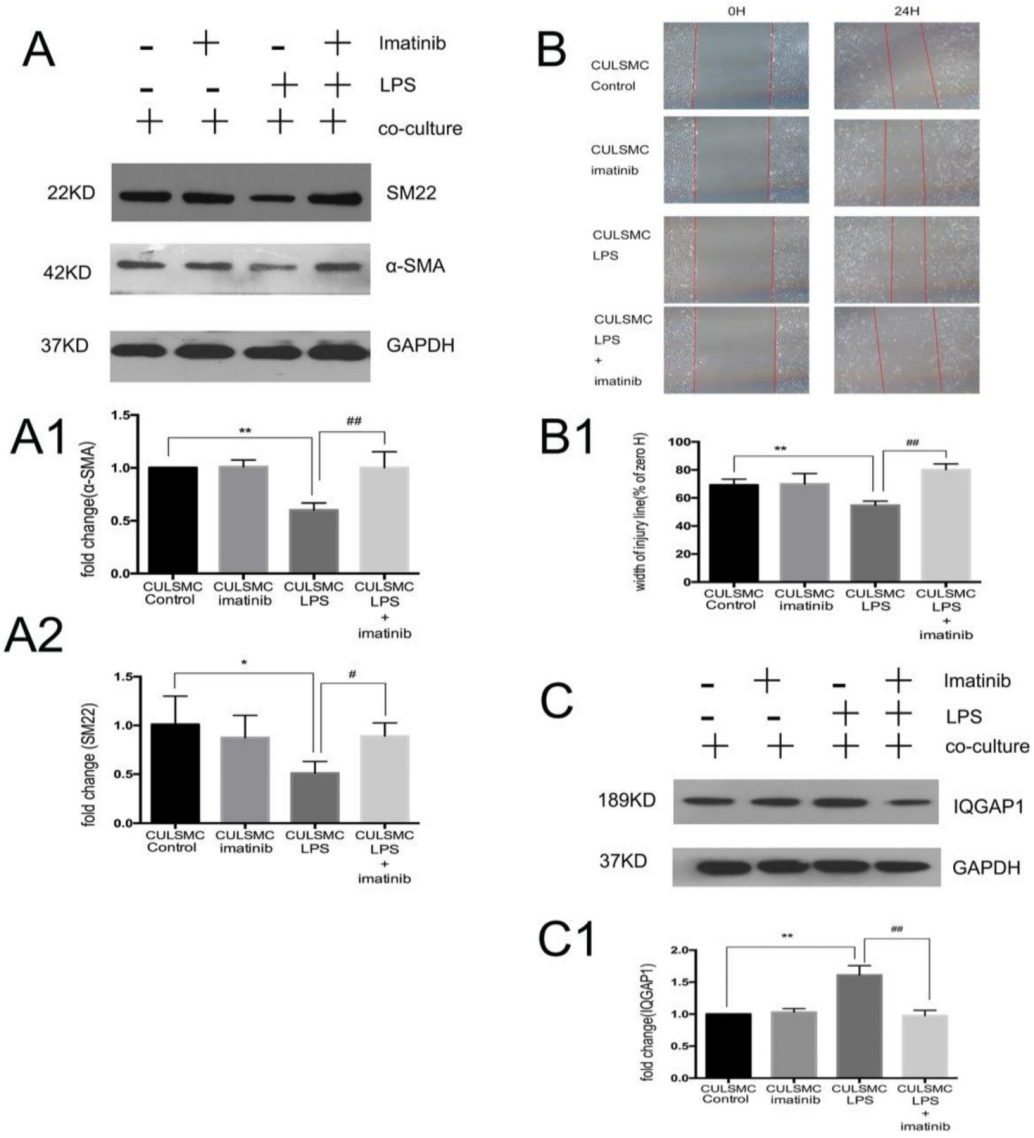
Imatinib attenuated vascular smooth muscle cell phenotype switching to a migration state by IQGAP1. **(A)**, The changes of α-SMA and SM22 expression were mediated by imatinib. Quantification of α-SMA is shown in A1; **: P<0.01, CULSMC Control *vs* CULSMC LPS, ##: P<0.01, CULSMC LPS *vs* SMCs were treated with LPS and imatinib in co-culture system (CULSMC LPS + imatinib). Quantification of SM22 expression is shown in A2; *: P<0.05, CULSMC Control *vs* CULSMC LPS, #: P<0.05, CULSMC LPS *vs* CULSMC LPS + imatinib; **(B)** LPS induced an increase in the migration ability of SMCs in the co-culture system were attenuated by imatinib. Quantification of B is shown in B1, **: P<0.01, CULSMC Control *vs* CULSMC LPS, ##: P<0.01, CULSMC LPS *vs* CULSMC LPS + imatinib; **(C)** The change of IQGAP1 expression was mediated by imatinib. Quantification of IQGAP1 is shown in C1; **: P<0.01, CULSMC Control *vs* CULSMC LPS, ##: P<0.01, CULSMC LPS *vs* CULSMC LPS + imatinib.

SMCs co-cultured in the absence of LPS were treated with imatinib (Figure 3A). Imatinib had no effect on the levels of p-PDGFR-β; however, as a PDGFR antagonist, the effects of imatinib on LPS induced migratory ability in SMCs. The phenotypic transition of SMCs and expression of IQGAP1 in the co-culture system were measured. We used imatinib at 5 μM for the following experiments; this concentration was confirmed by CCK8 assay (Figure S1). In Figure 4, the scratch-wound assay and western blotting showed that LPS induced a decrease in the expression of α-SMA and SM22, and an increase in the migration ability of SMCs in the co-culture system; these effects were attenuated by the PDGFR-β antagonist (Figure 4A and 4B). Next, IQGAP1 protein expression was determined in the absence and presence of imatinib. The results showed that LPS induced increased expression of IQGAP1 in SMCs, and imatinib prevented this increase in expression (Figure 4C).

## Discussion

The most characteristic aspects of sepsis may be its complex pathophysiological processes, in which vascular events are considered prominent; the role of ECs in vascular events has been expanded immensely 18-20. However, SMCs are another component of blood vessel with a less well-defined role during sepsis, and the relationship between ECs and SMCs has not yet been characterized.

A growing body of literature is indicating that SMCs exhibit considerable phenotypic plasticity 21-23. When vascular damage occurs, constricted SMCs are responsible for vascular contraction and expansion, and can be transformed into synthetic SMCs, which are characterized by decreased expression of SM22 and α-SMA 21, 24, 25 and increased migration ability. To date, few studies have reported on the involvement of ECs in transformation of the SMC phenotype during sepsis in a co-culture system.

In the present study, LPS treatment resulted in a decrease in the expression of contractile proteins and an increased migration ability in single-culture SMCs, and co-culture of ECs and SMCs resulted in alleviation of the expression of contractile proteins and suppression of migration ability in the presence or absence of LPS. Together, our results provide evidence that LPS induces SMC injury, and that ECs could partly reverse the change in the phenotypic transition and migration of SMCs.

PDGF is a potent mitogen for cells of mesenchymal origin, including fibroblasts, smooth muscle cells, and glial cells 26, 27. In both mouse and human, the PDGF signaling network consists of five ligands, PDGF-AA through -DD (including -AB), and two receptors, PDGFR-α and PDGFR-β. In general, expression of PDGFRs is low * in vivo*, but increases dramatically during inflammation. In human pulmonary alveolar epithelial cells, LPS has been shown to induce p42/p44 MAPK activation via the PDGFR/PI3K/Akt pathway 28. Previous research has revealed that PDGFR-β engages several well-characterized signaling pathways known to be involved in multiple LPS-induced cellular and developmental responses 29-31. Moreover, Oison et al. found that PDGFR-β signaling is involved in the differentiation of vascular smooth muscle 21. Similarly, in the present study, when compared to control groups, LPS induced a higher level of p-PDGFR-β in SMCs in both the single and co-culture systems. In co-culture SMCs, imatinib had no effect on p-PDGFR-β levels and could reverse the LPS-mediated higher level of p-PDGFR-β in SMCs.

A previous study found PDGFR-β could be suppressed by TNF-α 32, which is often involved in sepsis, and that TNF-α could reduce cell proliferation in response to PDGF-BB 33. In the present study, TNF-α only exhibited a slight upward trend, while the expression of PDGFR-β was significantly increased following treatment with LPS, which may be affected by multiple factors related to sepsis. In the absence of LPS stimulation, ECs promoted an increase in TNF-α expression in SMCs and tended to slightly decrease PDGFR-β expression; with LPS stimulation, there was an obvious increase in TNF-α expression and a decrease in PDGFR-β expression. Thus, TNF-α/PDGFR-β may be involved in the interaction between ECs and SMCs in co-culture, especially with LPS stimulation.

PDGF-BB is the highest affinity ligand for PDGFR-β. In a recent study involving a co-culture system, LPS-activated microglia stimulated PDGF-BB expression, enhanced angiogenesis, migration, proliferation, and permeability, and altered the phenotype of co-cultured renal microvascular endothelial cells 34. Previous studies have demonstrated that PDGF can be induced in ECs in response to injury or stimulus and play a vital role in SMCs and vessel remodeling 35, 36. However, no statistical differences were observed in PDGF-BB levels between the different groups in this study. We can speculate that not all PDGF family members were detected, only PDGF-BB. A similar hypothesis was proposed by Kim and colleagues; they determined that PDGF-BB levels did not respond to LPS treatment, but that PDGF-AA increased in a dose-dependent manner with LPS stimulation 37. Therefore, we suggest that the change in PDGFR-β was affected by TNF-α rather than PDGF-BB in our experiment.

In fact, a large corpus of data has shown that PDGFR-β participates in SMC migration 38, 39. Kohno and colleagues suggested that IQGAP1 might contribute to SMC migration through interaction with PDGFR-β 16. IQGAP1 mediates protein-protein interactions with a myriad of binding sites that regulate numerous signaling pathways, contributing to its multiple domains. For example, IQGAP1 forms scaffolds with several components of the Akt 15 and ERK 40, 41 pathways to facilitate diverse cellular functions. Further evidence has suggested that Akt signaling may regulate the SMC phenotype 23, and that IQGAP1 operates at both the leading and trailing edge of migrating cells via the ERK pathway 16. These results prompted us to further explore the regulation of IQGAP1 in response in injury and to determine whether IQGAP1 was involved in SMC differentiation. Consistent with the findings of previous studies 21, 42, increased expression of IQGAP1 was observed in LPS-treated SMCs in both the single and co-culture systems. Expression of IQGAP1 was higher in single culture compared with co-culture SMCs. In the co-culture system, the increased expression of IQGAP1 in LPS-treated SMCs was partly alleviated by the PDGFR-β inhibitor imatinib. When SMCs were treated with imatinib, the decreased expression of α-SMA and SM22, and LPS-induced increased migration of co-cultured SMCs, were attenuated. Thus, we conclude that LPS could induce the phenotypic transition of SMCs to migratory SMCs in the co-culture system via the TNF-α/PDGFR-β/IQGAP1 pathway, and that the different levels of these parameters in the single and co-culture systems may be due to the interaction between ECs and SMCs.

## Conclusions

Our findings indicate that the PDGFR-β/IQGAP1 pathway is involved in the interaction between ECs and SMCs, and that TNF-α may regulate PDGFR-β, especially in vascular injury with LPS stimulation. Thus, the interaction between ECs and SMCs serves an important role in vascular homeostasis and remodeling during sepsis. Indeed, this pathway may be a new target for the treatment of vascular damage that occurs with sepsis. Further study of additional signaling pathways involved in EC-mediated SMC phenotypic transformation will be necessary.

## Supplementary Material

Supplementary figure S1.
