# Supplementary figures and images for: The phenotype of vascular smooth muscle cells co-cultured with endothelial cells is modulated by PDGFR-β/IQGAP1 signaling in LPS-induced intravascular injury
Source: Int J Med Sci. 2019 Aug 6;16(8):1149–56. doi: 10.7150/ijms.34749 (PMC6743276; doi:10.7150/ijms.34749)

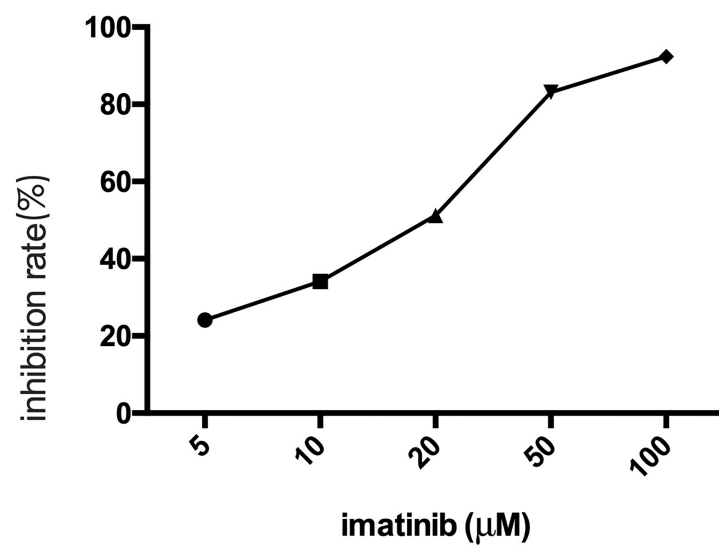

Supplement: Supplementary file 1 — Supplementary figure S1. [file ijmsv16p1149s1.pdf]
